# Prevalence and trigger factors of functional gastrointestinal disorders among male civil pilots in China

**DOI:** 10.1038/s41598-021-81825-0

**Published:** 2021-01-21

**Authors:** Chen Li, Junrong Xu, Daiwen Yin, Yuhai Zhang, Dezhi Shan, Xun Jiang, Lei Shang

**Affiliations:** 1 Department of Health Statistics, School of Preventive Medicine, Fourth Military Medical University, No.169 Changlexilu Road, Xi’an, 710032 Shaanxi People’s Republic of China; 2 Department of Gastroenterology, The Affiliated Hospital of Northwest University Xi’an No.3 Hospital, No.10 Eastern Section of the Third Fengcheng Road, Xi’an, 710032 Shaanxi People’s Republic of China; 3 Department of Pediatrics, Tangdu Hospital, Fourth Military Medical University, No 1 Xinsi Road, Xi’an, 710032 Shaanxi People’s Republic of China

**Keywords:** Gastrointestinal diseases, Functional gastrointestinal disorders, Psychology and behaviour

## Abstract

Functional gastrointestinal disorders (FGIDs) are common among the aircrew due to their arduous working environment. This study investigated the prevalence of FGIDs in Chinese male pilots and assessed the effects of trigger factors on the FGIDs. A cross-sectional study including 212 male pilots was performed in a Chinese large civil airline company. FGIDs were diagnosed according to the Rome IV diagnostic criteria. The psychological performance, dietary pattern, sleep situation, and physical activity of the respondents were assessed. Logistic regression analysis and structural equation modeling were used to explore the association between these trigger factors and FGIDs. FGIDs were observed in 83 (39.22%) respondents, of which 31 (37.35%) had overlap syndromes. Age, flight level, flight time, high-salt food pattern, anxiety, and sleep performance were found to be associated with FGIDs (all *P* < 0.05). Stepwise logistic regression analysis revealed that the flight level (OR 0.59, 95% CI 0.31–0.080), high-salt food pattern (OR 2.31, 95% CI 1.28–4.16), and sleep performance (OR 2.39, 95% CI 1.11–5.14) were the influencing factors associated with FGIDs. Structural equation modeling confirmed the correlations between FGIDs and the occupational, dietary, and psychological factors with a reasonable fit. The preventive strategies were necessitated according to occupational and psychological characteristics.

## Introduction

Functional gastrointestinal disorders (FGIDs) are characterized by chronic or recurrent gastrointestinal (GI) symptoms except for organic lesions, which include irritable bowel syndrome (IBS), functional dyspepsia (FD), functional heartburn(FH), functional constipation (FC), and so on^1^. An epidemiological study reported that 10–20% of the world population was affected by FGID^2^, and 1–8% of the FGIDs patients had two or three overlap syndromes^3^. These functional disorders rendered a negative impact on the quality of life of patients and caused a high disease burden, especially on some arduous occupational persons^4–6^. For example, a survey involving 1217 Korean firefighters reported that 37.9% of the respondents were diagnosed with FGIDs^4^. A United States’ study revealed that 76.8% of soldiers in Operation Iraqi Freedom and 54.4% of soldiers in Afghanistan experienced diarrhea^7^.

Commercial airplane pilots are also exposed to a high risk of suffering from FGIDs. The specific occupational environment including cosmic radiation and jet fuel^8^, long and irregular working patterns^9^ as well as crossing multiple time zones^10^ are their common occupational conditions^11^. Travel fatigue and disturbance of circadian rhythm can break the balance of their private life and induce various health problems^12,13^. Besides, occupational stress and emotional trauma are also experienced commonly among aircrews. A cross-sectional study on the Chinese air force population reported that 23.5% of 4630 initial participants were identified with more than one FGID, and the prevalence in the 787 aircrew members was as high as 20.33%^14^.

Besides the physical trauma, the dietary pattern is also closely associated with the onset and exacerbation of the FGIDs in pilots^15,16^. The diet types of pilots were relatively simple, unbalanced and unscheduled, which may also result in the risk of digestive disorder. On the other hand, psychological factors play an important role in the onset of FGID symptoms^17^. Previous studies revealed that among the patients with FGIDs, 30% presented with possible depression while 22% presented with possible anxiety^18^. The symptoms of FGIDs were severe when accompanied by depression, anxiety, and other psychiatric disorders^19^. In case of aviator occupation, the flight environment, vibration, and noise may lead to anxiety, occupational stress, and other emotional problems. However, pilots with FGIDs may always overlook the importance of psychosocial factors^14,20,21^.

Until now, only a few studies reported the risk of FGIDs that the pilots faced. Moreover, few study measured the prevalence of FGIDs for Chinese civil pilots and the possible mechanisms between the trigger factors and the development of FGIDs. Given the gender differences on the FGIDs epidemiology^22,23^ and low proportion of female among civil pilots in China^24^, this study aimed to investigate the prevalence of FGIDs among the commercial male pilots in China with the new Rome IV diagnostic criteria and assess the effects of the physical and mental performance on the FGIDs, so as to optimize the health care management during their career.

## Methods

### Participants

The study was conducted in accordance with the principles of the Declaration of Helsinki. The Ethics Committee of the Fourth Military Medical University carefully considered and approved the project proposal. Between November 2018 and December 2018, representative male pilot employees in a large civil airline company in China were recruited using a convenience sampling methodology, and a cross-sectional survey was performed. Male pilots, aged 20–50 years, who had taken sick leave for no more than 4 weeks during the recruitment period were eligible for participation. The pilots with organic esophageal or GI tract disease were excluded. Informed consent was obtained from all individual participants included in the study. All the respondents were assured that no names were registered in the questionnaires and their answers would not impact on their occupational achievement or their future work.

### Measures

After being interviewed by flight surgeons, all respondents were required to complete a self-report questionnaire, which consisted of the following parts: part 1 focused on demography and job description such as age, body mass index (BMI), marital status, education, and so on; part 2 included the Rome IV diagnostic criteria^25^ to evaluate the FGIDs; part 3 assessed the psychological performance using the self-rating anxiety scale (SAS) and self-rating depression scale (SDS)^26,27^; part 4 assessed the dietary patterns of participants with three-point semi-quantitative food frequency questionnaire(SQFFQ),; and part 5 evaluated the sleep and physical activity situations of pilots using the Pittsburgh sleep quality index (PSQI^28^ and the International Physical Activity Questionnaire Short Form (last7-self)^29^. All the responses were recorded on one computer using the EPIDATA 3.1 data documentation software.

### FGID

The diagnosis of FGID was based on the new Rome IV criteria^25^. The questionnaire for Rome IV diagnostic criteria was translated to Chinese according to Chinese culture. Eighty pilots were randomly selected to complete the Chinese questionnaire in order to assess its comprehension and accuracy. The internal consistency reliability estimates for each section were within the desired range with Cronbach’s $\alpha$ coefficient 0.82–0.89. After two times surveys with the kappa coefficient of 0.83, the Chinese questionnaire was applied to diagnose the FGID. If one of the FGID manifestations was identified, the respondent was diagnosed with FGIDs. The overlap syndrome was defined as at least two of the FGID symptoms. According to the manifestations of FGID, the respondents were divided into three groups: the healthy group, the single FGID group, and the combined symptom FGID group.

### Diet

The semi-quantitative food frequency questionnaire was designed to collect the food list, frequency of consumption and the portion size consumed^30^. Referenced a lot of Chinese food frequency questionnaire^31–33^, we selected 27 food categories often eaten in Chinese dietary, which covered cereals, meat, vegetables, fruits, egg products, nuts, fish, and so on. There was also an open question to detect the subject’s favor food which not contained in the given 27 food categories. The intake frequencies were classified into eight levels: almost never; less than one time per month; 1–3 times per month; one times per week; 2–4 times per week; 5–6 times per week; one time per day; 2–3 times per day and 4 or more times per day. There was no unified portion size in China, and therefore, food types and portion size of local food were surveyed with visual aids, which consisted of photographs of utensils and food portions to assist with description of amounts consumed. Options for the average portion sizes were 0.5, 1, 1.5, 2. Based on the food composition and food model^34,35^, it investigated the food intake of each subject in the recent half year. And a 3-day 24-h dietary recalls (24 h DRs) was also used to evaluate the reliability and validity of this SQFFQ.

The diet pattern was identified by exploration factor analysis^36^. The common factors with Eigenvalues > 1.0 were extracted based on the screen plot. An orthogonal rotation procedure was applied to simplify the factor structure and render it more easily interpretable. The derived factors were named as the different dietary patterns according to the food categories that loaded most strongly on the factor. For each participant, the factor score for each pattern was calculated by summing the quantity of each food grouping weighted by their loading on each factor. The dietary pattern scores were expressed as three quartiles for comparison across levels of intake.

### Psychology

The evaluations related to psychology, sleep, and physical activity were performed according to the corresponding standard criteria. Specifically, the respondents with the SAS index scored above 50 and the SDS index scored above 0.50 were defined as having anxiety and depression, respectively^26,27^. A PSQI score of 1–5 was considered as sleep quality, 6–10 as sleep latency, 11–15 as sleep duration, and 15–21 as sleep disturbance^28^. The physical activity intensity was divided as low (the total physical activity score below 600), median (the total physical activity score between 600 and 3000), and high (the total physical activity score above 3000)^29^.

### Statistical analyses

Statistical analyses were performed using SAS 9.1.3 (SAS Institute Inc., USA). Continuous variables were expressed by mean ± standard deviation when the data was approximately normally distributed, otherwise a non-parametric alternative should be used instead. The categorical variables were expressed by numbers and percentages. A one-way ANOVA was used to compare the normal distribution variables, followed by a LSD test for post hoc analysis to further examine the differences among the groups. The chi-square test was used to test the differences for categorical variables. Stepwise logistic regression analysis was conducted to investigate the associations between various factors and FGIDs. Structural equation modeling (SEM), using M-Plus (Muthen & Muthen, Mplus, Version 7), was applied to explore the associations between latent physical and mental factors and the developing of FGIDs. A two-tailed *P* value of less than 0.05 was considered to be statistically significant.

## Results

### Characteristics of participants

A total of 212 male pilots were sampled in this cross-sectional study. All the participants completed their questionnaire with a response rate of 100%. As shown in Table 1, the respondents demonstrated a mean age of 33.83 ± 7.05 years (range 22–48 years), a BMI of 23.84 ± 2.35 kg/m^2^ (range 18.94–29.00 kg/m^2^), and 87.74% of them were Han race. The proportion of smokers and alcohol drinkers was 48.83% and 31.92%, respectively. More than 40% of the respondents were the captain and (senior) first officer. The mean flight time was 19.62 ± 10.11 h within 1 month, with the day-night flight ratio as 2:1. Of all the pilots, almost 50% had no long voyage within 1 month, and 16.98% had long voyages three or four times every month. Approximately 80% of the respondents reported that they had undergone a regular physical examination 6 months prior and had no disease history.

**Table 1:** Demographic characteristic of the study participants.

Characteristics	Description
Age (years)	33.83 ± 7.05
BMI (kg/m^2^)	23.84 ± 2.35
**Race**	
Han	186 (87.74)
Minority	26 (12.26)
**Marital status**	
Single	44 (20.75)
Married	168 (79.25)
**Education**	
University	210 (99.02)
Postgraduate	2 (0.98)
**Alcohol**	
No	123 (57.75)
Yes	68 (31.92)
Cessation	21 (9.86)
**Smoke**	
No	101 (47.41)
Yes	104 (48.83)
Cessation	7 (3.29)
**Flight level**	
Captain	25 (11.79)
First officer	77 (36.32)
Second officer	42 (19.81)
Pilot cadet	68 (32.08)
**Flight hours within 1 month**	
< 10	52 (24.51)
10–19	89 (42.16)
≥ 20	71 (33.33)
**Day flight hours within 1 month**	
< 10	104 (49.02)
≥ 10	108 (50.98)
**Night flight hours within 1 month**	
< 10	170 (80.39)
≥ 10	42 (19.61)
**Long voyage (≥ 8 h) frequency within 1 month**	
0	105 (49.53)
1–2	71 (33.49)
3–4	36 (16.98)

Continuous variables were expressed by mean ± standard deviation and categorical variables were expressed by numbers (percentages).

### Spectrum of FGID

From a total of 212 respondents, 83 (39.15%) were identified with FGIDs, four major categories of FGIDs were observed based on the Rome IV criteria: bowel disorders (20.28%), gastroduodenal disorders (17.45%), esophageal disorders (13.68%) and central mediated disorders of gastrointestinal pain (0.94%) (Table 2).

**Table 2:** Types of functional gastrointestinal disorders with Rome IV diagnostic criteria.

Type^a^	n	% of all respondents	% of FGIDs
**Any FGID**	83	39.15	100
**A. Esophageal disorders**	29	13.68	34.94
A2. Functional heartburn	6	2.83	7.23
A3. Functional chest pain	8	3.77	9.64
A4. Globus	15	7.08	18.07
B**. Gastroduodenal disorders**	37	17.45	44.58
B1. Functional dyspepsia	28	13.21	33.73
B3. Nausea and vomiting disorders	7	3.30	8.43
B4. Rumination syndrome	2	0.94	2.41
**C. Bowel disorders**	43	20.28	51.81
C1. Irritable bowel syndrome	20	9.43	24.10
C2. Functional constipation	9	4.25	10.84
C3. Functional diarrhea	6	2.83	7.23
C4. Functional abdominal bloating	8	3.77	9.64
**D. Central mediated disorders of gastrointestinal pain**	2	0.94	2.41
D1. Central Mediated abdominal pain syndrome	2	0.94	2.41
**Combined FGIDs**	31	14.62	37.35
Two-way combination	18	8.49	21.69
A + B	4	1.89	4.82
A4 + B3	4	1.89	4.82
A + C	4	1.89	4.82
A3 + C1	2	0.94	2.41
A4 + C1	2	0.94	2.41
B + C	10	4.72	12.05
B1 + C1	4	1.89	4.82
B1 + C4	4	1.89	4.82
B1 + C2	2	0.94	2.41
**Three-way combination**	7	3.30	8.43
A + B + C	7	3.30	8.43
A4 + B1 + C1	4	1.89	4.82
A4 + B1 + C2	3	1.42	3.61
**Four-way combination**	6	2.83	7.23
A + B + C	6	2.83	7.23
A3 + A4 + B1 + C1	2	0.94	2.41
A2 + A3 + B1 + C1	2	0.94	2.41
A2 + B4 + C1 + C3	2	0.94	2.41

^a^The serial numbers of A–D were coded according to the Rome IV criteria.

The prevalence of individual FGID is as follow. Globus (7.08%), Functional dyspepsia (FD: 13.21%), and IBS (9.43%) were the most frequent diagnoses among bowel disorders, gastroduodenal disorders and esophageal disorders respectively, whereas FC (4.25%), functional chest pain (FCP: 3.77%), and functional abdominal bloating (FAB: 4.72%) also had a moderate prevalence. Only two aviators had rumination syndrome, and two other aviators had central-mediated abdominal pain syndrome.

**Figure 1:**
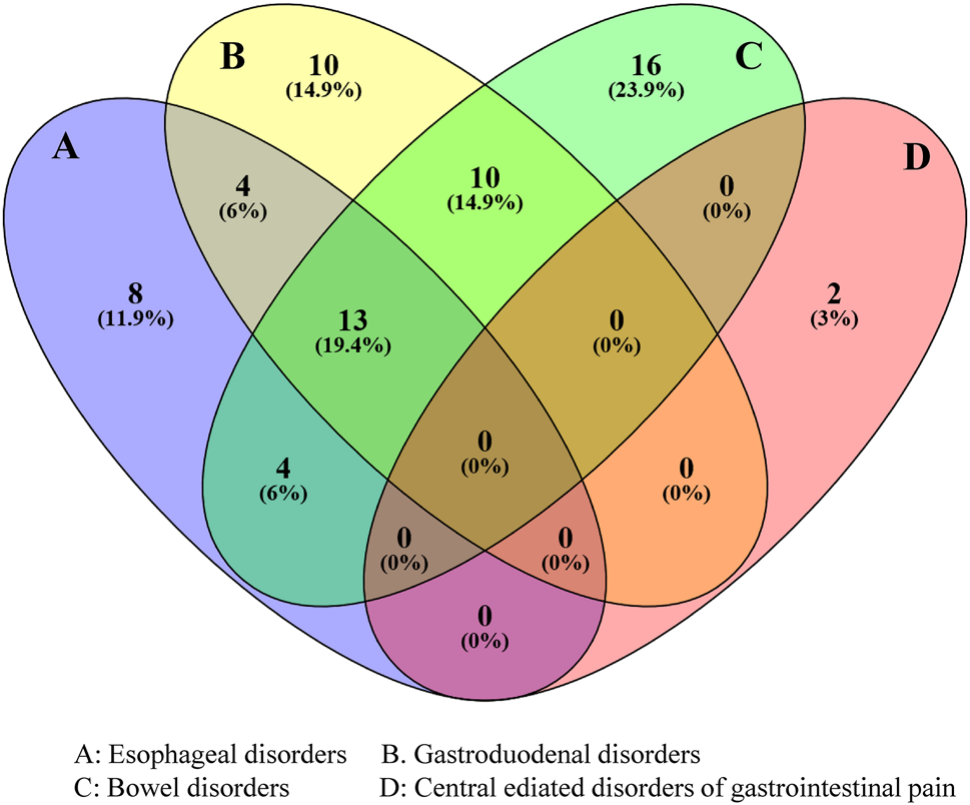
Venn diagram of the combined FGIDs between esophageal, gastroduodenal, bowel and anorectal disorders in the respondents based on Rome IV criteria.

As for the overlap syndrome, 31 pilots met the criteria for more than one FGID symptom. The categories of FGIDs occurred in different combinations were shown as two-way combination 8.49%, three-way combination 3.33% and four-way combination 2.83% (Table 2 and Fig. 1). Bowel disorders and gastroduodenal disorders were the most prevalent (4.72%) in the two-way combination, whereas central mediated disorders of gastrointestinal pain had no combination. For the detailed syndrome, globus + nausea and vomiting disorders (NVD), FD + IBS and FD + FAB led the two-way combination (1.89%). Among the seven pilots with three-way combined FGIDs (4.72%), four were diagnosed with FD + IBS + globus. While, the six pilots with four-way overlap syndromes all manifested IBS syndromes.

### Diet pattern

The SQFFQ demonstrated acceptable relative validity and high reproducibility with intraclass correlation coefficients (ICCs) of 0.44–0.77, spearman’s correlation coefficients of 0.35–0.62 and the three-point quartile agreement of 72.0–95.0% in the same or adjacent quartiles for the food categories referenced 24 h DRs. Via the three-point semi-quantitative food frequency questionnaire, four dietary patterns were retained for analysis as follow: the vegetarian pattern characterized by green vegetables and fruits, the high-salt food pattern characterized by Chinese pickles and bacon, the starch food pattern characterized by the Chinese traditional staple food of rice and noodles and the protein food pattern characterized by high-quality protein products such as fish, milk and mushrooms.

The dietary pattern scores were expressed as three quartiles for comparing the levels of intake between different FGID groups, as shown in Table 3. The vegetarian pattern, the starch food pattern and protein food pattern were not related to the distribution of FGIDs. However, respondents with high-salt food pattern scores above 66.7% were more likely to be identified with FGIDs than those with lower scores (*P* < 0.001). The odds ratio [OR 2.46, 95% confidence level (CI) 1.71–3.35] indicated that high-salt food pattern was positively associated with FGIDs; for every 33% increase in the high-salt food pattern score, the risk of FGIDs might double.

**Table 3:** Food patterns for different FGID groups.

Dietary pattern^a^	No FGID (%) (n = 129)	Single FGID (%) (n = 52)	Combined FGIDs (%) (n = 31)	*P*	OR (95% CI)
**Vegetarian pattern**					
≤ P_33.3_	52 (40.32)	23 (41.67)	17 (53.33)	0.41	
P_33.3_–P_66.6_	35 (27.42)	17 (33.33)	8 (26.67)		
> P_66.6_	42 (32.26)	12 (25.00)	6 (20.00)		
**High-salt food pattern**					
≤ P_33_	59 (45.16)	12 (25.00)	2 (6.67)	< 0.01	2.46 (1.71, 3.55)^a^
P_33_–P_66_	39 (30.65)	19 (37.50)	10 (33.33)		
> P_66_	31 (24.19)	21 (37.50)	19 (60.00)		
**Starch food pattern**					
≤ P_33_	48 (37.10)	27 (51.92)	12 (38.71)	0.40	
P_33_–P_66_	50 (38.71)	13 (25.00)	11 (35.48)		
> P_66_	31 (24.19)	12 (23.08)	8 (25.80)		
**Protein food pattern**					
≤ P_33_	50 (38.71)	29 (55.77)	10 (32.26)	0.12	
P_33_–P_66_	48 (37.10)	12 (23.08)	10 (32.26)		
> P_66_	31 (24.19)	11 (21.15)	11 (35.48)		

^a^The reference is the low-level group.

### ***Psychological situation***

According to the evaluation criteria of the SAS^27^, the anxiety scores were different between the three groups, as shown in Table 4 (*P* = 0.006). The overlap syndrome group had the highest anxiety score (48.75 ± 13.30), followed by the single FGID syndrome group (44.48 ± 6.46). In the post hoc analysis, the anxiety score was significantly higher in the overlap syndrome group than in the healthy group, which meant that the more FGID syndromes the respondents had, the higher anxiety scores they got.

**Table 4:** Comparison the psychological scoring among the participants.

Variable scoring	No FGID (n = 129)	Single FGID (n = 52)	Combined FGIDs (n = 31)	*P*	Post hoc^a^
Anxiety	40.65 ± 8.61	44.48 ± 6.46	48.75 ± 13.30	< 0.01	1 < 3
Depression	0.42 ± 0.13	0.46 ± 0.11	0.51 ± 0.18	0.09	
Physical strength	2859.85 ± 2400.93	2883.21 ± 3081.83	3380.95 ± 2131.82	0.82	
Sleep	10.26 ± 2.84	11.78 ± 3.31	11.27 ± 2.82	< 0.01	1 < 2

^a^Post hoc analysis was used LSD test, 1,2, AND 3 represents no FGID group, Single FGID group and Combined FGIDs group, respectively.

The depression and physical activity situation showed no significant difference between the three groups. According to the criteria of PSQI (short version), the single syndrome group had the highest sleep score (11.78 ± 3.31), followed by the combined syndrome group (11.27 ± 2.82) and the healthy control group (10.26 ± 2.84) (*P* = 0.005). The score of the single syndrome group was significantly higher than that of the control group.

### Associations between predictor variables and FGIDs

Table 5 shows a number of variables significantly associated with the prevalence of FGIDs. The respondents over 35 years were more likely to have FGIDs. For the occupation factors, the flight levels were adversely affected by the FGIDs. The captain had the highest prevalence of FGIDs, followed by the first and second officer. The aircrew flying for more than 20 h every month had almost one times higher prevalence of FGIDs than those flying for less than 10 h.

**Table 5:** Factors associated with functional gastrointestinal disorders.

Characteristics	No FGID (%) (n = 129)	Any FGIDs (%) (n = 83)	*P*	OR (95% CI)
**Age (years)**				
< 35	89 (70.08)	38 (29.92)	< 0.01	
≥ 35	40 (47.06)	45 (52.94)		
**Race**				
Han	111 (59.68)	75 (40.32)	0.35	
The other	18 (69.23)	8 (30.77)		
**BMI (kg/m**^**2**^**)**				
18.5–24.9	80 (66.13)	68 (69.88)	0.35	
≥ 25	39 (33.87)	25 (30.12)		
**Marital status**				
Single	30 (71.43)	12 (28.57)	0.12	
Married	99 (59.24)	71 (41.76)		
**Education**				
University	129 (61.43)	81 (38.57)	0.07	
Postgraduate	0	2 (100.00)		
**Alcohol**				
No	79 (64.23)	44 (35.77)	0.41	
Yes	37 (54.51)	31 (45.59)		
Cessation	13 (61.90)	8 (38.10)		
**Smoke**				
No	62 (61.39)	39 (38.61)	1.00	
Yes	63 (60.58)	41 (39.42)		
Cessation	4 (57.14)	3 (42.86)		
**Flight level**				
Captain	10 (40.00)	15 (60.00)	< 0.01	0.59 (0.31, 0.80)
First officer	42 (54.55)	35 (45.45)		
Second officer	23 (54.76)	19 (45.24)		
Pilot cadet	54 (79.41)	14 (20.59)		
**Flight time per month**				
< 10 h	37 (71.15)	15 (28.85)	0.03	
10–19 h	57 (64.04)	32 (35.96)		
≥ 20 h	35 (49.30)	36 (50.70)		
**Long voyage frequency per month**				
0	62 (59.05)	43 (40.95)	0.29	
1–2 times	48 (67.61)	23 (32.39)		
3–4 times	19 (52.78)	17 (47.22)		
**Vegetarian pattern**				
< P_33.3_	52 (56.52)	40 (43.48)	0.22	
P_33.3_–P_66.6_	35 (58.33)	25 (41.67)		
> P_66.6_	42 (70.00)	18 (30.00)		
**High-salt food pattern**				
< P_33.3_	59 (80.82)	14 (19.18)	< 0.01	2.31 (1.28, 4.16)
P_33.3_–P_66.6_	39 (57.35)	29 (42.65)		
> P_66.6_	31 (43.66)	40 (56.34)		
**Starch food pattern**				
< P_33.3_	48 (55.17)	39 (44.83)	0.27	
P_33.3_–P_66.6_	50 (67.57)	24 (32.43)		
> P_66.6_	31 (60.78)	20 (39.22)		
**Protein food pattern**				
< P_33.3_	50 (56.18)	39 (43.82)	0.26	
P_33.3_–P_66.6_	48 (68.57)	22 (31.43)		
> P_66.6_	31 (58.49)	22 (41.51)		
**Anxiety**				
Yes	8 (32.00)	17 (68.00)	< 0.01	
No	121 (64.71)	66 (35.29)		
**Depression**				
Yes	39 (54.17)	33 (45.83)	0.15	
No	90 (64.29)	50 (35.71)		
**Physical activity**				
Low	15 (75.00)	5 (25.00)	0.39	
Median	49 (59.76)	26 (40.24)		
High	65 (59.09)	45 (40.91)		
**Sleep performance**				
Sleep quality (PSQI score: 1–5)	6 (50.00)	6 (50.00)	< 0.01	2.39 (1.11, 5.14)
Sleep latency (PSQI score: 6–10)	52 (80.00)	13 (20.00)		
Sleep duration (PSQI score: 11–15)	69 (53.49)	60 (46.51)		
Sleep disturbance (PSQI score: 16–21)	2 (33.33)	4 (66.67)		

^a^OR was obtained from stepwise logistic regression analysis.

The high-salt food pattern was positively associated with the development of FGIDs. The prevalence of the upper three-quartile score group was almost three times of those in the lower three-quartile score group (56.34% vs 19.18%), which meant that the respondents with high-salt food pattern were more likely to have FGIDs.

The psychological factors and the FGIDs were also related. The prevalence of FGIDs in anxious aircrews was two times than that in aircrews without anxiety (*P* = 0.002). The depression and physical strength did not correlate with the FGIDs, but a good sleep quality might positively prevent the occurrence of FGIDs. The pilots with sleep scores of 16–21 had the highest prevalence of FGIDs, which was approximately three times than the prevalence in those with good sleep.

The multivariate logistic regression analysis showed that three predictors were found to independently and significantly correlate with the FGIDs: flight level (OR 0.59; 95% CI 0.31–0.80; *P* = 0.001), high-salt food pattern (OR 2.31; 95% CI 1.28–4.16; *P* < 0.001), and sleep score level (OR 2.39, 95% CI 1.11–5.14; *P* = 0.002) (Table 5, Model chi-square test, *P* < 0.001; Hosmer–Lemeshow’s goodness-of-fit test, *P* < 0.001).

We also explored a subgroup analysis of the risk factors by FGID types. Given the relatively small number for each specific FGID symptom, this analysis was mainly conducted on the sub-type FGIDs as the esophageal disorders, gastroduodenal disorders, bowel disorders according to Rome IV diagnostic criteria. As shown in Table 6, the trigger factors for the subgroup syndromes were quite similar to those for FGIDs. For example, the higher flight level pilots with anxiety symptoms were more likely to have esophageal disorders. While, age, flight level, flight time per month, and high-salt food pattern were found to associate with the gastroduodenal disorders significantly. The older age, the high flight level, the high-salt food pattern, the anxious psychology and the poor sleep performance might be the potential factors contributing to the bowel disorders.

**Table 6:** Factors associated with functional gastrointestinal disorders.

Characteristics	Esophageal disorders (%^a^) (n = 29)		Gastroduodenal disorders (%^a^) (n = 37)		Bowel disorders (%^a^) (n = 43)	
**Age (years)**						
< 35	16 (12.60)	0.15	16 (12.60)	< 0.01	16 (12.60)	< 0.01
≥ 35	13 (15.29)		25 (29.41)		27 (31.76)	
**Race**						
Han	25 (13.44)	0.98	30 (16.13)	0.46	39 (20.97)	0.43
The other	4 (15.38)		7 (26.92)		4 (15.38)	
**BMI (kg/m**^**2**^**)**						
18.5–24.9	24 (16.22)	0.10	28 (18.92)	0.33	29 (19.59)	0.98
≥ 25	5 (7.81)		9 (14.06)		14 (21.88)	
**Marital status**						
Single	3 (7.14)	0.12	4 (9.52)	0.09	9 (21.43)	0.75
Married	26 (15.29)		33 (19.41)		34 (20.00)	
**Education**						
University	29 (13.81)	–	36 (17.14)	0.22	42 (20.00)	0.25
Postgraduate	0		1 (50.00)		1 (50.00)	
**Alcohol**						
No	14 (11.38)	0.38	18 (14.63)	0.13	24 (19.51)	0.75
Yes	12 (17.65)		17 (25.00)		15 (22.06)	
Cessation	3 (14.29)		2 (9.52)		4 (19.05)	
**Smoke**						
No	9 (8.91)	0.14	18 (17.82)	1.00	20 (19.80)	0.78
Yes	20 (19.23)		19 (18.27)		23 (22.12)	
Cessation	0		1 (14.29)		2 (28.57)	
**Flight level**						
Captain	6 (24.00)	< 0.05	6 (24.00)	0.01	6 (24.00)	0.01
First officer	9 (11.69)		17 (22.08)		20 (25.97)	
Second officer	8 (19.05)		9 (21.43)		11 (26.19)	
Pilot cadet	6 (8.82)		5 (7.35)		6 (8.82)	
**Flight time per month**						
< 10 h	7 (13.46)	0.51	7 (9.62)	0.03	11 (21.90)	0.78
10–19 h	11 (12.36)		15 (16.85)		18 (21.13)	
≥ 20 h	11 (15.49)		17 (23.94)		14 (13.89)	
**Long voyage frequency per month**						
0	13 (12.38)	0.73	15 (14.29)	0.22	23 (21.90)	0.79
1–2 times	10 (14.08)		12 (16.90)		15 (21.13)	
3–4 times	6 (16.67)		10 (27.78)		5 (13.89)	
**Vegetarian pattern**						
< P_33.3_	10 (10.87)	0.30	13 (14.13)	0.07	20 (21.74)	0.21
P_33.3_–P_66.6_	12 (20.00)		17 (28.33)		15 (25.00)	
> P_66.6_	7 (11.67)		7 (11.67)		8 (13.33)	
**High-salt food pattern**						
< P_33.3_	8 (10.96)	0.06	6 (8.22)	< 0.01	5 (6.85)	< 0.01
P_33.3_–P_66.6_	8 (11.76)		14 (20.59)		16 (23.53)	
> P_66.6_	13 (18.31)		17 (23.94)		22 (30.99)	
**Starch food pattern**						
< P_33.3_	13 (14.94)	0.82	14 (16.09)	0.76	21 (24.14)	0.50
P_33.3_–P_66.6_	10 (13.51)		12 (16.22)		13 (17.57)	
> P_66.6_	6 (11.76)		11 (21.57)		9 (17.65)	
**Protein food pattern**						
< P_33.3_	12 (13.48)	0.89	14 (15.73)	0.71	21 (23.60)	0.10
P_33.3_–P_66.6_	10 (14.29)		12 (17.14)		9 (12.86)	
> P_66.6_	7 (13.21)		11 (20.75)		13 (24.53)	
**Anxiety**						
Yes	6 (24.00)	0.01	6 (24.00)	0.05	10 (40.00)	< 0.01
No	23 (12.30)		31 (16.58)		33 (17.65)	
**Depression**						
Yes	11 (15.28)	0.42	17 (23.61)	0.07	20 (27.78)	0.05
No	18 (12.86)		20 (14.29)		23 (16.43)	
**Physical activity**						
Low	1 (7.69)	0.42	2 (15.38)	0.56	3 (23.08)	0.57
Median	12 (19.35)		15 (24.19)		15 (24.19)	
High	16 (15.24)		20 (19.05)		25 (23.81)	
**Sleep performance**						
Sleep quality	1 (8.33)	0.06	2 (16.67)	0.08	4 (33.33)	0.01
Sleep latency	5 (7.69)		8 (12.31)		8 (12.31)	
Sleep duration	22 (17.05)		25 (19.38)		28 (21.71)	
Sleep disturbance	1 (16.67)		2 (33.33)		3 (50.00)	

^a^The prevalence of the sub-type FGID in the corresponding classification. *P* values were based on χ^2^ test between the sub-type FGID group and no-FGOD group.

### Structural equation model

A confirmatory factor analysis (CFA) using Structural equation model (SEM) was conducted to assess the linear association between the effects of the trigger factors on the FGIDs. Age, flight factors, food categories with a factor loading of more than 0.4 in the high-salt food pattern, and psychological factors were considered to be enrolled in the analysis. The resulting SEM is illustrated in Fig. 1, in which the chi-square test result was statistically significant (*χ*^2^ = 223.72, *P* < 0.001), the comparative fit index was (0.97) > 0.90, the root-mean-square error of approximation was (0.02) < 0.06, and the degree of freedom ratio was (1.03) < 3.00.

**Figure 2:**
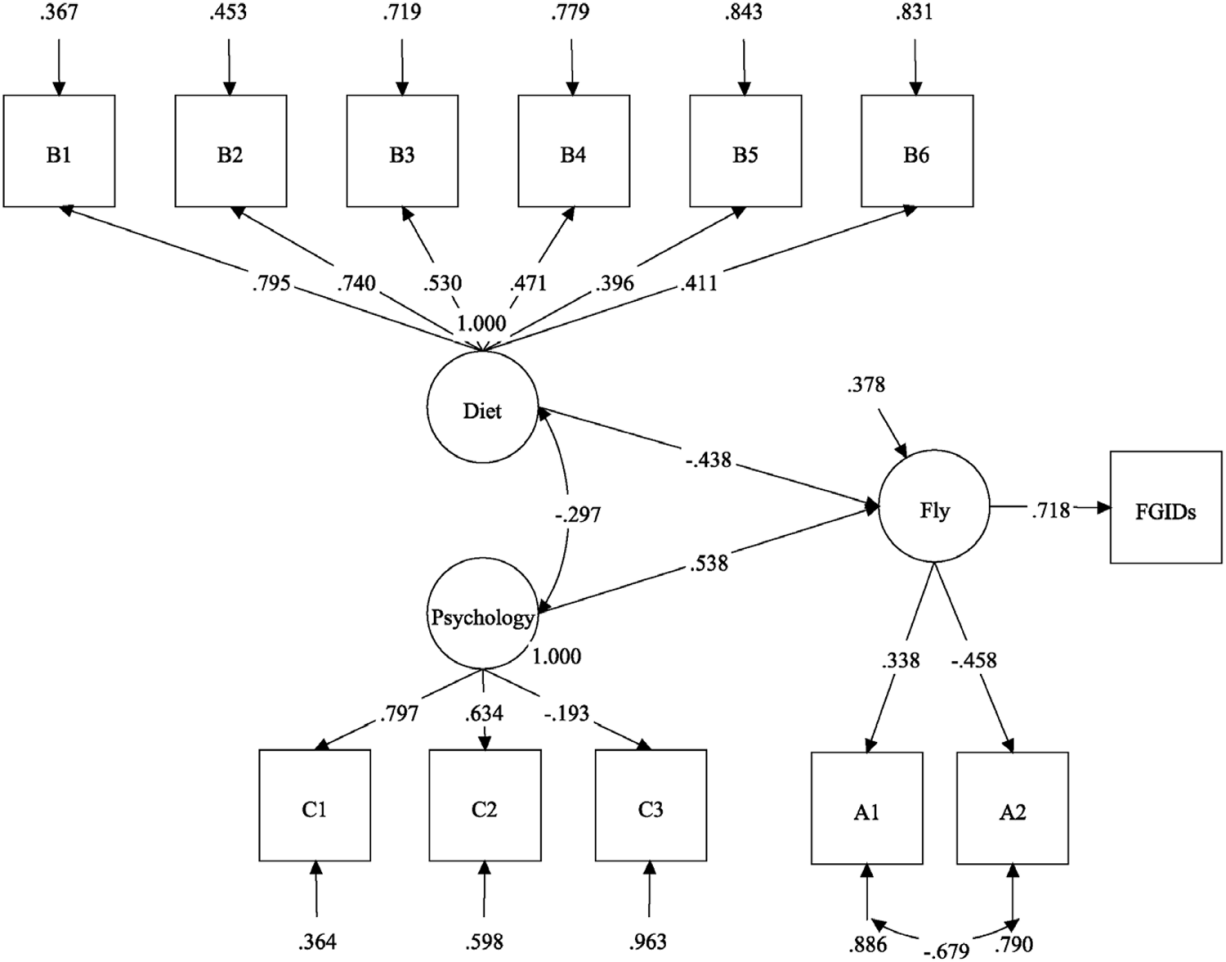
Parameter estimates (standard regression coefficients) of the structure equation model for the effects of the trigger factors on the FGIDs. A1: age, A2: flight level, B1-B6: six food categories with factor loading > 0.4 in the high-salt food pattern, which were B1: chicken, B2: animal giblets, B3: Chinese pickles, B4: water food (one of Chinese pickled vegetable), B5: preserved meat and B6: bacon, C1: anxiety, C2: depression, C3: sleep performance.

As Fig. 2 indicated, the diet pattern and the latent psychology variable were negatively associated with each other (standardized regression coefficient, − 0.30), which meant that less high-salt food might be related to good psychology. Both of them affected the latent flying variable with $\beta =$  − 0.44 and $\beta =$ 0.54, respectively, indicating that the aviators with high flying level might prefer the high-salt food and endure poorer psychology. Finally, the latent flying variable positively correlated with FGIDs ($\beta =$ 0.72). For the explanatory power of the SEM, the diet and psychology accounted for approximately 50% variance in the flying variable, which resulted in 71.80% of the variance in the FGIDs, indicating that the presence of other latent factors might be linked to FGIDs.

## Discussion

As studies on the prevalence of FGIDs in Chinese aviators were very limited, this study was conducted to identify the effect of the trigger factors on the FGIDs for the civil pilots. We have just focused on the FGIDs of male pilots. That is because the prevalence of FGIDs varied substantially between female and male^37^, such as FD^22,37,38^ and IBS^23,39,40^. While, the proportion of female in Chinese pilots was very low, which was reported as only 1.85% for the commercial certificate pilots and 0.33% for the aviation transport license pilots^24^. In this study, we had used the convenience sampling methodology to recruited the subjects. There were too few female pilots attending this research voluntarily in the recruiting phase. On the contrary, female mostly undertake the cabin crews in China. Although both of the pilots and cabin crew are exposed to the same working environment, the potential activity level and occupational variables are quite varied. Considering the gender and occupational difference, we do not include the female pilots in this study. Therefore, it should be cautious when generalizing the results to the characteristics of all the aircrews.

In this study, 39.15% of the respondents had FGIDs. This value was high compared with that in some other studies, in which the prevalence of military aircrews was 7.2% in the USA^41^ and 3.50% in China^14^. However, this was lower than those reported by Park (49.7%)^42^ and Trivedi in their study including soldiers presenting to a home hospital (50%)^43^. The diversity in prevalence might be because the first survey on the Chinese commercial pilots used Rome IV criteria. It might also be because of the developments in the Chinese aviation and tourism industries, in which the aviator occupation required a highly efficient and error-free performance. The pilots undergo very stressful physical and psychiatric training, which may be the reason of the high prevalence of FGIDs among them. Although the pilots with organic esophageal or GI tract disease were excluded in this survey, the helicobacter pylori status were not evaluated among the participants, which were also an essential for differential diagnosis.

This study also found that FD was the most prevalent FGIDs overall (13.21%), followed by IBS (9.43%) and globus (7.08%). The main subtype rank was similar to that in other studies, such as Bang^44^, Wu^14^, and Dong^45^. However, the prevalence in this study was quite higher than these studies, which were reported as 5.85% and 4.04% for FD and IBS for the Chinese air forth pilots’ prevalence in Wu’s research^14^. It also showed a higher prevalence of FD and IBS than the general populations, in which the global prevalence of FD was reported as 6.9% (95 CI 5.7–8.2) from a meta-analysis in 2020 comprising 81,144 subjects from 4 studies with the ROME IV criteria^22^. Another meta-analysis comprising 82,476 individuals from 34 countries claimed the pooled IBS prevalence was 3.8% (95 CI 3.1–4.5) used the Rome IV criteria^23^. Although researches suggested the Rome IV criteria might be stricter for the diagnostic of FGIDs compare to the prior criterion^46,47^, the prevalence of FD and IBS in this study were higher than those in the systematic reviews. Therefore, cautious interpretations should be made regarding this aspect.

In terms of overlapping syndromes, the prevalence was estimated as 14.62% of the total population and 37.35% of the patients with FGIDs in this study. In previous studies, the prevalence was reported to range from 1 to 17%^3,48–51^, not much different from the results of this study. As for the most combination syndromes, this study showed that FD + IBS, FD + FAB, and NVD + globus (1.89%) were the most prevalent subtype constitutions, which was quite similar to the findings of studies on healthy military males from the USA^52^ and Korea^53^.

However, it's worth noting that previous studies had found that there may be few difference for Rome IV criteria compared to the prior Rome criterion^46,54^. For example, a study recruited 1375 adults self-identified as having IBS found that the Rome IV criteria significantly under reported the prevalence of IBS in comparison to Rome III(59.1% vs. 78.9%)^47^. Some systematic review also reported that the pooled prevalence of uninvestigated dyspepsia and IBS were lower with Rome IV(dyspepsia: 6.9% vs. 11.5%; IBS: 3.8 vs. 9.2%)^22,23^. It seemed that Rome IV criteria were more restrictive. The agreement between Rome III and Rome IV is still needed to be detected further. Understanding the impact of these changes to the diagnostic classification system for FGIDs will be important in the future.

In addition, the results also found several trigger factors related to FGIDs. They included occupational exhaustion, dietary pattern, psychological stress, and sleep performance. For the occupational environment, high-speed flying, constant exposure to rapid acceleration and deceleration, and noise and vibration might be the complicated mechanisms resulting in abdomen discomfort^55–57^. Therefore, the flight level and flight time were closely associated with the development of FGIDs.

Similar to other studies^57,58^, the effects of dietary patterns on the FGIDs were proved by the CFA and SEM results. Although carbohydrates, proteins, and individual fatty acids were the most frequently compounds that influenced the digestive functions, a positive association was found between the high-salt dietary pattern and the FGIDs in this study. The high-salt intake might be of particular importance for the composition and activity of intestinal microbiota^59^. Specific nutrients could change the microbial metabolic activity, leading to GI discomfort.

In agreement with the results of previous studies, psychological distress was found to be positive related to FGIDs and combined syndromes in our research. The anxiety factor was proved to be an independent risk factor for FGIDs, whereas no statistical correlation was observed between depression and FGIDs. This result was similar to a study of the brain–gut pathway^60^, but not coincident with some studies, which found that depression had a negative correlation with FGIDs^14,44^. The exact reason or mechanism was unclear. More studies are needed to elucidate the association between each psychological factor and FGIDs.

In conclusion, this is the first population-based survey using Rome IV criteria to evaluate FGIDs among Chinese pilots, which has found that there is a high overall prevalence of FGIDs among Chinese male pilots, especially on FD, IBS and Globus. The SEM analysis elaborated the effects of the flight factor, food pattern, psychology and sleep performance on the FGIDs. Therefore, further works can be conducted on more integrative prevention and treatment combining psychological and physical approaches for the developing and progression of FGIDs.

This study also had some limitations. First, it could not identify a causal relationship between some influence factors and FGIDs, because it was a cross-sectional study with subjective bias. Second, the information was obtained using self-administered questionnaires, in which may conceal a possibility of subjective bias. Third, only male aviators were included in this study, generalizing the results to the characteristics of all commercial pilots with FGIDs was difficult. More researches are required on a wide range of respondents and the various trigger factors in the future.

## Data Availability

The datasets used and/or analyzed during the current study are available from the corresponding author on reasonable request.
